# Obesity, Metabolic Health, and Diabetic Complications in People With Type 1 Diabetes

**DOI:** 10.1002/edm2.70017

**Published:** 2024-12-16

**Authors:** Yuanjie Mao, Jen‐Tzer Gau, Ning Jiang

**Affiliations:** ^1^ Diabetes Institute Ohio University Athens Ohio USA; ^2^ Endocrinology Clinic OhioHealth Castrop Health Center Athens Ohio USA; ^3^ Department of Primary Care, Heritage College of Osteopathic Medicine Ohio University Athens Ohio USA; ^4^ Primary Care and Geriactrics OhioHealth Primary Care and Geriatrics Athens Ohio USA; ^5^ Cardiology Clinic LifeBridge Health Cardiovascular Institute Westminster Maryland USA

**Keywords:** cardiovascular events, complications, metabolic health, obesity, type 1 diabetes mellitus

## Abstract

**Aim:**

The concept of metabolically healthy obesity (MHO) has not been studied in type 1 diabetes (T1D). By analysing datasets from the DCCT/EDIC study, we compared the development of diabetic complications by obesity and metabolic health over 30 years of follow up.

**Materials and Methods:**

Insulin resistance was calculated by estimated glucose disposal rate (eGDR). The participants (*n* = 1127) were then divided into four groups based on time‐weighted mean body mass index and mean eGDR: metabolically healthy non‐obesity (MHN, *n* = 874), metabolically unhealthy non‐obesity (MUN, *n* = 66), MHO (*n* = 146) and metabolically unhealthy obesity (MUO, *n* = 41). Diabetic complications and cardiovascular events were compared across the four groups.

**Results:**

MUO and MUN groups had significantly higher risk for peripheral neuropathy (*p* = 0.001 in MUO and *p* < 0.001 in MUN vs. MHN), cardiac autonomic neuropathy (*p* < 0.001 in both MUO and MUN vs. MHN), retinopathy (*p* = 0.001 in MUO and *p* < 0.001 in MUN vs. MHN) and microalbuminuria (*p* < 0.001 in both MUO and MUN vs. MHN) than MHN group. Moreover, MUO and MUN groups had significantly higher risks (HR [95%CI]) in any cardiovascular events (2.78 [1.51–5.11] and 1.88 [1.05–3.36]) and major atherosclerotic cardiovascular events (2.72 [1.16–6.37] and 2.31 [1.05–5.10]) compared to MHN group. However, the risk of these complications and cardiovascular events (except peripheral neuropathy and cardiac autonomic neuropathy) in MHO group was not different from that in MHN group.

**Conclusions:**

This study highlights the importance of metabolic health represented by insulin resistance in the development of diabetic complications and cardiovascular events in T1D beyond their weight status.

## Graphical abstract

Obesity, metabolic health, and the development of diabetic complications in type 1 diabetes.

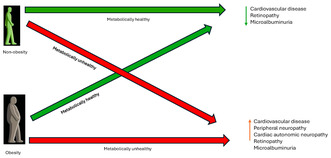

## 1 Introduction

Obesity has become a worldwide epidemic that poses substantial health and economic burden for both individuals and community. Obesity is often associated with a complex of metabolic abnormalities, including prediabetes, type 2 diabetes, hypertension, atherogenic dyslipidaemia, non‐alcoholic fatty liver disease and cardiovascular diseases [1, 2, 3]. Obesity also contributes to impaired quality of life, disabilities and reduced life expectancy [2]. However, findings from many previous studies showed that a subgroup of individuals with obesity might be protected from major adverse metabolic effects of excess body fat or might be at substantially lower risk than expected for their extent of adiposity. This subgroup has been described as having metabolically healthy obesity (MHO) [4, 5]. This novel concept of MHO becomes increasingly important to stratify individuals in the clinical management of obesity.

Individuals with MHO are those who meet the standard body mass index (BMI) cutoff point for obesity (≥ 30 kg/m^2^) but without other major cardiovascular risk factors [2]. Individuals with major cardiovascular risk factors are consequently judged as people with metabolically unhealthy obesity (MUO). However, there is no universally accepted definition of MHO. Most studies define MHO as having either 0, 1 or 2 components of metabolic syndrome, while many others define MHO as insulin‐sensitive based on the homeostasis model assessment of insulin resistance [2].

This concept of MHO has not been studied in people with type 1 diabetes (T1D), although recent studies have shown that close to 50% of people with T1D were overweight or obese [6]. In the present study, we define MHO by insulin sensitivity measured by estimated glucose disposal rate (eGDR), which is calculated using routine clinical measures: haemoglobin A1c (HbA1c), waist‐ to‐ hip ratio and the presence of hypertension [7, 8, 9, 10]. The euglycemic‐hyperinsulinemic clamp is the standard for measurement of insulin resistance; however, it is not practical in the clinical setting. Since endogenous insulin secretion in T1D is negligible, several derived indexes from clamp study have been proposed to represent insulin resistance. The eGDR showed good correlation with insulin resistance measured by the euglycemic‐hyperinsulinemic clamp in T1D [7, 8] and has been validated for the estimation of insulin sensitivity in diverse populations including in the DCCT/EDIC and the Pittsburgh Epidemiology of Diabetes Complications study cohorts [9, 10]. It showed a better predictor value for diabetic complications than metabolic syndrome, total daily insulin dose and other insulin resistance estimations [6, 9, 10, 11]. By analysing datasets from the DCCT/EDIC study, we compared the development of diabetic complications and cardiovascular events by their obesity and metabolic health status over 30 years of follow up in people with T1D.

## 2 Materials and Methods

### 2.1 Participants

A detailed description of DCCT/EDIC study has been published [12, 13]. In brief, DCCT (1983–1993) was a controlled clinical trial of 1441 patients with T1D randomised to conventional diabetes therapy or intensive therapy to assess whether reducing hyperglycaemia would decrease the risk of complications of T1D [12]. At baseline, these patients aged 13 to 39 years, had T1D for 1 to 15 years and were in generally good health. They had no proliferative retinopathy and no albuminuria (urinary excretion of albumin < 200 mg/24 h). Subjects with a history of cardiovascular diseases or with hypertension (blood pressure ≥ 140/90 mmHg) or hypercholesterolemia (fasting serum cholesterol > 3 SDs above age‐ and sex‐specific means) were not eligible to participate [14].

At the end of the DCCT, after 6.5 years of mean follow‐up, 1375 of the 1425 surviving members (96%) of the original cohort volunteered to participate in the long‐term EDIC follow‐up study starting in 1994 [13]. During EDIC, all therapy was provided by the patients' own physicians, and intensive therapy was recommended for all patients. The current report includes follow‐up data of 78% of the surviving participants (*n* = 1127) with full information from DCCT entry through EDIC year 20 (2013). BMI was calculated every year during the follow up, and insulin resistance was estimated using the following index at baseline and each year in EDIC [6]: eGDR: 24.31 – (12.22 × waist‐to‐hip ratio) – (3.29 × hypertension [defined as 0 = no, 1 = yes]) – (0.57 × HbA1c [%]). Hypertension was defined as systolic blood pressure ≥ 140 mmHg or diastolic blood pressure ≥ 90 mmHg or the use of anti‐hypertensive agents for the treatment of hypertension. We adopted BMI ≥ 30 as the cut off for obesity, and eGDR < 5.6 as the cut off for insulin resistance since it comes from a previous study in the U.S. population which is consistent with our study cohort [8, 15]. The participants were then divided into four groups based on their time‐weighted mean BMI and time‐weighted mean eGDR over the 30 years of follow‐up: metabolically healthy non‐obesity (MHN) group (insulin sensitive and not obese, *n* = 874), metabolically unhealthy non‐obesity (MUN) group (insulin resistant and not obese, *n* = 66), metabolically healthy obesity (MHO) group (insulin sensitive and obese, *n* = 146) and metabolically unhealthy obesity (MUO) group (insulin resistant and obese, *n* = 41). The de‐identified data sets were acquired from the National Institute of Diabetes and Digestive and Kidney Diseases Central Repository. This research protocol was approved by Ohio University research ethics committee.

### 2.2 Biomedical Evaluations

During EDIC, participants had an annual history update, physical examination and laboratory testing, using the same standardised methods employed during the DCCT [16]. HbA1c was measured by high‐performance liquid chromatography quarterly during DCCT and annually during EDIC. Fasting lipid profiles and 4‐h urine collections for measurement of albumin excretion rate and creatinine clearance were measured annually during DCCT and on alternate years during EDIC [13]. The DCCT/EDIC central biochemistry laboratory performed all laboratory measurements with standardised methods and long‐term controls to minimise variation.

In EDIC year 8 (2000), serum adiponectin levels were measured by using a radioimmunoassay procedure developed by Linco Research, and C‐reactive protein (CRP) levels were determined by high sensitivity nephelometry (Nephelometer 100, Dade Behring, Marburg, Germany). Some covariates in our analyses were fixed, whereas others were time‐dependent co‐variates representing the updated mean of all follow‐up values since randomisation. For co‐variates measured at different frequencies during DCCT and EDIC (e.g., BMI and eGDR), the updated time‐weighted mean was calculated weighting each value by the interval between measurements.

### 2.3 Carotid Artery and Cardiac Imaging

Carotid intima‐media thickness (IMT) measurement was performed in EDIC year 12 (2004) as described [17]. A single longitudinal lateral view of the distal 10 mm of the right and left common carotid arteries were obtained by certified technicians at the clinical centres and read in a central unit by two readers [18]. The maximum IMT (millimetre) of the common carotid artery was defined as the mean of the maximum IMT for near and far walls on both right and left sides.

Cardiac magnetic resonance imaging was performed to measure heart structure and function with a uniform protocol during EDIC year 14 to 16 [19]. Coronary artery calcification assessment was performed during EDIC year 7 to 9. The methods to obtain the Computed tomography‐derived calcium scores were previously described [14, 20]. All participants were scanned twice over calibration phantoms of known physical calcium concentration. The measurement of coronary artery calcium was assessed by measuring all pixels with density > 130 Hounsfield units. The calcium score was obtained by summing all calcific lesions in all four major coronary arteries and side branches. The average score from the two scans was used in the analysis [20]. Coronary artery calcification was defined as the calcium score > 0 and the severe coronary artery calcification was defined as the calcium score > 200 [20].

### 2.4 Outcomes

The primary cardiovascular disease (CVD) outcome was time to the first occurrence of any of the following types of cardiovascular events: cardiovascular death, nonfatal myocardial infarction (MI), nonfatal stroke, subclinical MI on electrocardiogram, angina confirmed by ischemic changes with exercise tolerance testing or by clinically significant obstruction on coronary angiography, revascularisation (with angioplasty or coronary artery bypass), or congestive heart failure (paroxysmal nocturnal dyspnoea, orthopnoea, or marked limitation of physical activity caused by heart disease) [21]. The secondary CVD outcome, major atherosclerotic cardiovascular events (MACE), a subset of CVD events, included only the time to cardiovascular death, nonfatal MI or nonfatal stroke, whichever occurred first [21]. The data were retrieved to maximal 30 years since entry.

Peripheral artery disease was defined by ankle brachial index ≤ 0.9 in EDIC year 19–20. Retinopathy was defined by the presence of proliferative diabetic retinopathy (PDR) and/or history of panretinal scatter photocoagulation (laser) therapy in EDIC year 19–20 [15]. Peripheral neuropathy was confirmed from nerve conduction testing in EDIC year 13 or 14 [19]. The presence of cardiac autonomic neuropathy (CAN) was evaluated using cardiovascular autonomic reflex tests that assessed the R‐R response to paced breathing (R‐R variation), the Valsalva manoeuvre and postural changes in blood pressure in EDIC year 13 or 14. CAN was defined as an R‐R variation < 15, or an R‐R variation 15–19.9 combined with a Valsalva ratio < 1.5, or a decrease of > 10 mmHg in diastolic blood pressure after standing for 10 min. The 66 subjects with CAN at baseline were excluded from these analyses [22]. Microalbuminuria was defined as a sustained albumin excretion rate of 30 mg per 24 h or higher on two consecutive study visits during DCCT and through EDIC year 19–20. ESRD included kidney transplantation or dialysis [14]. Chronic kidney failure was defined as ESRD or the development of an estimated glomerular filtration rate (eGFR) < 15 mL/min/1.73 m^2^ using the Chronic Kidney Disease Epidemiology Collaboration equation [23].

### 2.5 Statistical Analyses

The scale variables were expressed as mean ± SD except the CRP and adiponectin levels were log‐transformed because of the skewed distribution. The differences between four groups were analysed using Fisher's exact test or a one‐way ANOVA followed by the Tukey post‐hoc test for multiple comparisons. Multivariable logistic regression models were used to estimate odds ratios (ORs) and 95% confidence intervals (CIs) of association of the four groups with diabetic complications (peripheral artery disease, coronary artery calcification, retinopathy, peripheral neuropathy, cardiac autonomic neuropathy, microalbuminuria and chronic kidney failure). Adjustments were made for age, sex, duration of diabetes, treatment groups (conventional or intensive therapy) in DCCT and the presence of non‐proliferative retinopathy at baseline. Cox proportional hazards multivariate models assessed hazard ratios (HRs) and 95% CIs of the association of age, sex, duration of diabetes, treatment groups in DCCT, the presence of non‐proliferative retinopathy at baseline and the four groups with MACE and CVD. All statistical tests were two‐sided and P‐values of less than 0.05 were considered statistically significant. Statistical analyses were done by using SPSS 19 (IBM, Armonk, NY).

### 2.6 Data and Resource Availability

The data sets generated during and analysed during the current study are available in the National Institute of Diabetes and Digestive and Kidney Diseases Central Repository. No applicable resources were generated or analysed during the current study.

## 3 Results

### 3.1 Baseline Demographic and Clinical Characteristics of Participants by Weight and Metabolic Status

The demographic and clinical characteristics of participants by metabolic health and weight status were described in Table 1. Amongst the 1127 study participants, 146 (13.0%) were MHO, whereas 41 (3.6%) were MUO. Participants with MHO were more common in women, having higher time‐weighted mean BMI, more insulin resistance represented by lower time‐weighted mean eGDR, higher time weighted mean systolic and diastolic blood pressure, higher time‐weighted mean triglyceride and LDL cholesterol levels, and lower time‐weighted mean HDL cholesterol levels compared to those with MHN (Table 1). There was no difference in age, duration of diabetes, time‐weighted mean HbA1c, time‐weighted mean total daily insulin dose and time‐weighted mean total cholesterol between the MHO and MHN groups (Table 1). Besides, participants with MHO were younger, more in women, having shorter duration of diabetes, lower time‐weighted mean BMI, lower time mean‐weighted systolic and diastolic blood pressure, and lower time‐weighted mean triglyceride levels compared to those with MUO (Table 1).

**TABLE 1:** Baseline demographic and clinical characteristics of participants by weight and metabolic status.

	Metabolically healthy non‐obesity (MHN) *n* = 874	Metabolically unhealthy non‐obesity (MUN) *n* = 66	Metabolically healthy obesity (MHO) *n* = 146	Metabolically unhealthy obesity (MUO) *n* = 41	*p* for trend
Age (years)	49 ± 7	52 ± 6*	49 ± 7	54 ± 7*, ^&^	< 0.001
Female sex, *n* (%)	417 (47.7%)	10 (15.2%)*	89 (61.0%)*, ^#^	8 (19.5%)*, ^&^	< 0.001
Smoking at entry, *n* (%)	146 (16.7%)	19 (28.8%)*	16 (11.0%)^#^	8 (19.5%)	0.014
Ever smoking, *n* (%)	273 (31.2%)	30 (45.5%)*	39 (26.7%)^#^	14 (34.1%)	0.054
Duration of diabetes (Months)	68 ± 50	83 ± 50	57 ± 44^#^	81 ± 53^&^	0.001
Intensive therapy in DCCT, *n* (%)	435 (49.8%)	29 (43.9%)	87 (59.6%)*, ^#^	27 (65.9%)*, ^#^, ^&^	0.021
Non‐proliferative retinopathy at baseline, *n* (%)	434 (49.7%)	44 (66.7%)*	67 (45.9%)^#^	27 (65.9%)*, ^&^	0.007
Statin ever use, *n* (%)	652 (74.6%)	62 (93.9%)*	113 (77.4%)^#^	38 (92.7%)*, ^&^	< 0.001
ACE inhibitor ever use, *n* (%)	571 (65.3%)	64 (97.0%)*	107 (73.3%)^#^	38 (92.7%)*, ^&^	< 0.001
ARB ever use, *n* (%)	264 (30.2%)	44 (66.7%)*	59 (40.4%)*, ^#^	27 (65.9%)*, ^&^	< 0.001
Time‐weighted BMI (kg/m^2^)	25.3 ± 2.4	27.1 ± 2.0*	32.8 ± 2.5*, ^#^	33.0 ± 3.4*, ^&^	< 0.001
Time‐weighted eGDR	8.1 ± 1.3	4.8 ± 0.7*	7.5 ± 1.1*, ^#^	4.7 ± 0.6*, ^&^	< 0.001
Time‐weighted HbA1c (%)	7.9 ± 0.9	8.9 ± 1.0*	8.0 ± 0.9^#^	8.4 ± 1.0*	< 0.001
Time‐weighted Insulin dose/weight (units/kg)	0.62 ± 0.17	0.71 ± 0.17*	0.64 ± 0.18	0.68 ± 0.20	< 0.001
Time‐weighted systolic blood pressure (mmHg)	117 ± 8	127 ± 7*	120 ± 7*, ^#^	128 ± 7*, ^&^	< 0.001
Time‐weighted Diastolic blood pressure (mmHg)	74 ± 5	79 ± 5*	76 ± 4*, ^#^	79 ± 3*, ^&^	< 0.001
Time‐weighted total cholesterol (mg/dL)	180 ± 23	194 ± 23*	185 ± 27^#^	192 ± 20*	< 0.001
Time‐weighted triglyceride (mg/dL)	80 ± 37	120 ± 53*	92 ± 42*, ^#^	118 ± 62*, ^&^	< 0.001
Time‐weighted HDL cholesterol (mg/dL)	56 ± 13	48 ± 9*	52 ± 11*	49 ± 11*	< 0.001
Time‐weighted LDL cholesterol (mg/dL)	108 ± 20	122 ± 20*	114 ± 23*, ^#^	120 ± 18*	< 0.001

*Note:* Data are expressed by mean ± SD or *n* (%).

Abbreviations: ACE, Angiotensin‐converting enzyme; ARB, Angiotensin II receptor blockers; BMI, body mass index; eGDR, estimated glucose disposal rate; HbA1c, haemoglobin A1c; HDL, high‐density lipoprotein; LDL, low‐density lipoprotein; MHN, metabolically healthy non‐obesity; MHO, metabolically healthy obesity; MUN, metabolically unhealthy non‐obesity; MUO, metabolically unhealthy obesity.

* *p* < 0.05 compared to MHN group.

^#^ *p* < 0.05 compared to MUN group.

^&^ *p* < 0.05 compared to MHO group.

There were significantly higher rates of both smoking at entry and ever smoking in the MUN group, and lower rates in the MHO group, than that in the MHN group. No difference between MUO and MHN groups was found (Table 1). There were significantly more statin, Angiotensin‐converting enzyme inhibitors, and Angiotensin II receptor blockers ever users in the MUN and MUO groups than that in the MHN and MHO groups. Besides, there were more Angiotensin II receptor blockers ever users in the MHO group than in the MHN group (Table 1).

### 3.2 Inflammatory Markers by Weight and Metabolic Status

**FIGURE 1:**
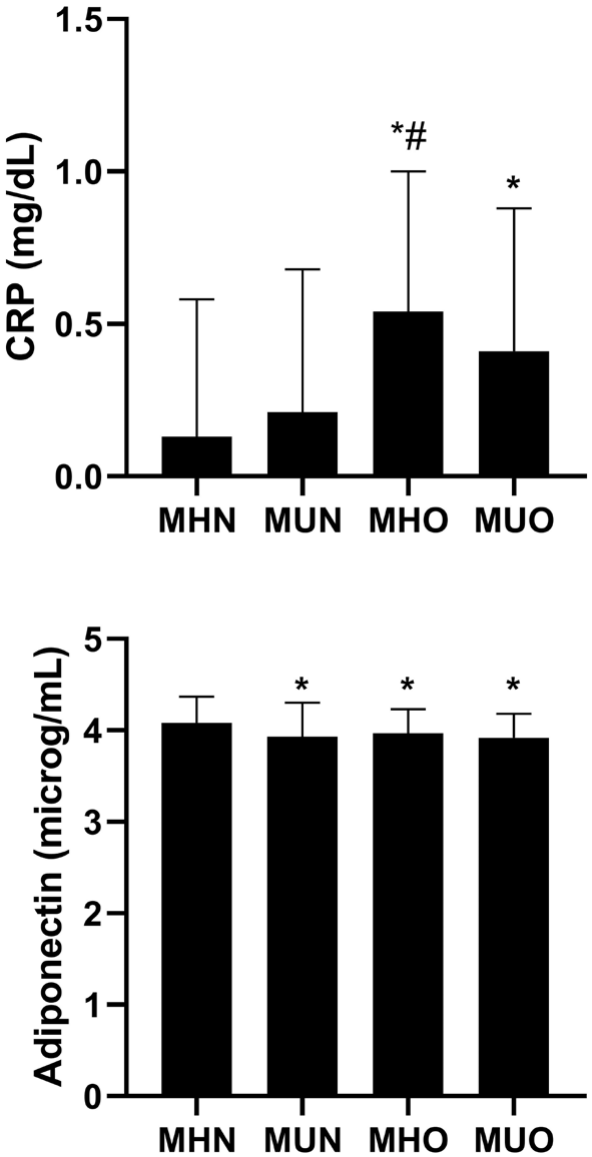
Serum CRP and adiponectin levels in the four groups. Data are presented as the log transformed mean ± S.D. *, *p* < 0.05 compared to MHN group; #, *p* < 0.05 compared to MUN group; *p* < 0.05 compared to MHO group. CRP, C‐reactive protein; MHN, metabolically healthy non‐obesity; MHO, metabolically healthy obesity; MUN, metabolically unhealthy non‐obesity; MUO, metabolically unhealthy obesity.

We next examined the serum CRP and adiponectin levels by weight and metabolic status (Figure 1). The results showed that participants with MHO and MUO had higher CRP and lower adiponectin levels than those with MHN. There was no difference of CRP and adiponectin levels between the MHO and MUO groups.

### 3.3 Diabetic Complications by Weight and Metabolic Status

**FIGURE 2:**
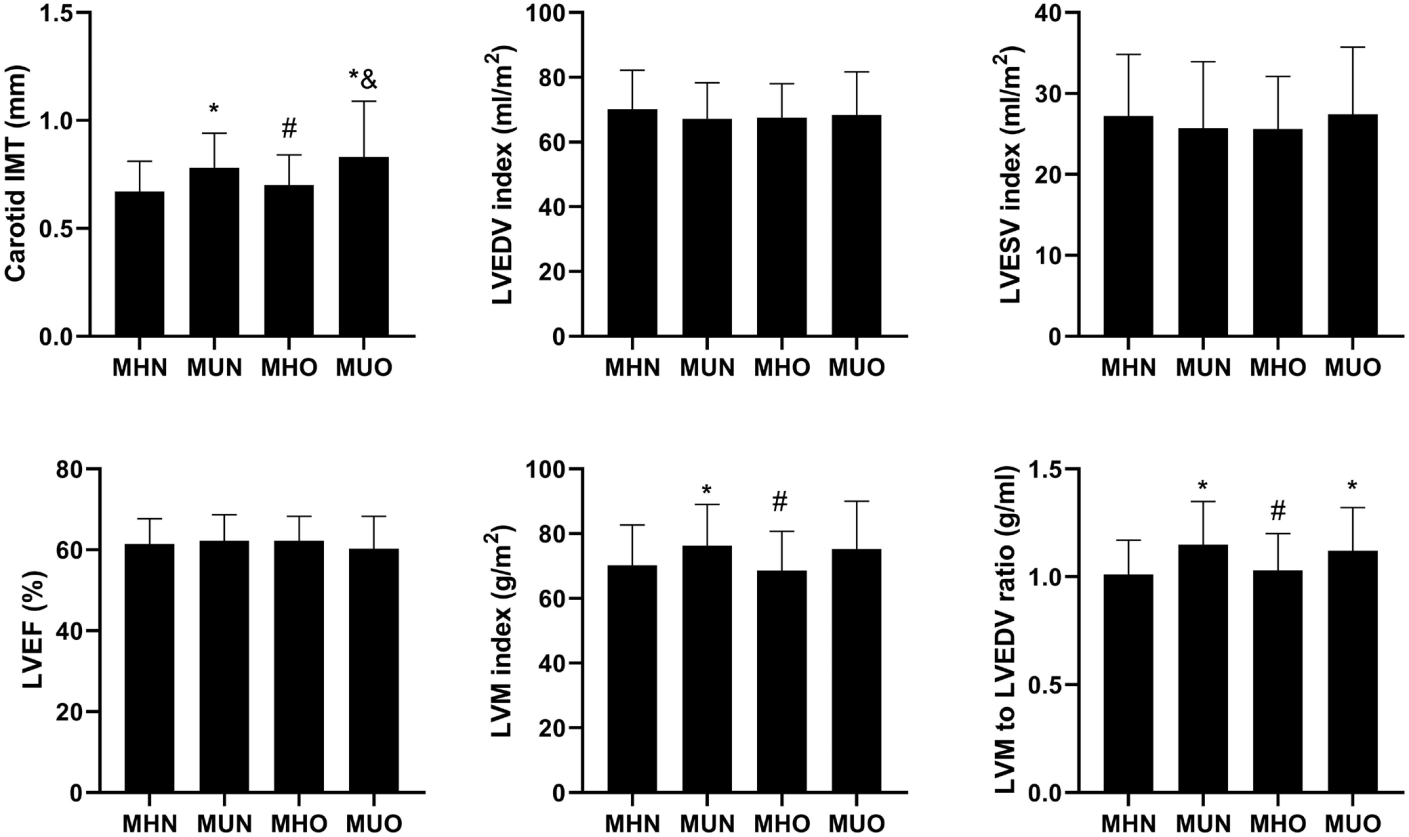
Carotid IMT and cardiac magnetic resonance imaging results. Data are presented as the mean ± S.D. *, *p* < 0.05 compared to MHN group; #, *p* < 0.05 compared to MUN group; &, *p* < 0.05 compared to MHO group. EDV, end‐diastolic volume; EF, ejection fraction; ESV, end‐systolic volume; IMT, intima‐media thickness; LV, left ventricle; LVM, left ventricular mass; MHN, metabolically healthy non‐obesity; MHO, metabolically healthy obesity; MUN, metabolically unhealthy non‐obesity; MUO, metabolically unhealthy obesity.

The difference of carotid IMT and cardiac magnetic resonance imaging results in the four groups were compared. The carotid IMT in the MHO group was significantly lower than that in the MUO and MUN groups (0.70 ± 0.14 vs. 0.83 ± 0.26 vs. 0.78 ± 0.16 mm, *p* < 0.001), while there was no statistical difference between the MHO and MHN groups (0.70 ± 0.14 vs. 0.67 ± 0.14 mm, *p* = 0.33). There was no difference in left ventricle end‐diastolic volume and end‐systolic volume, and left ventricle ejection fraction among the four groups. Left ventricle mass index and Left ventricle mass to end‐diastolic volume ratio of the MHO group was lower than those of MUN groups (Figure 2).

**FIGURE 3:**
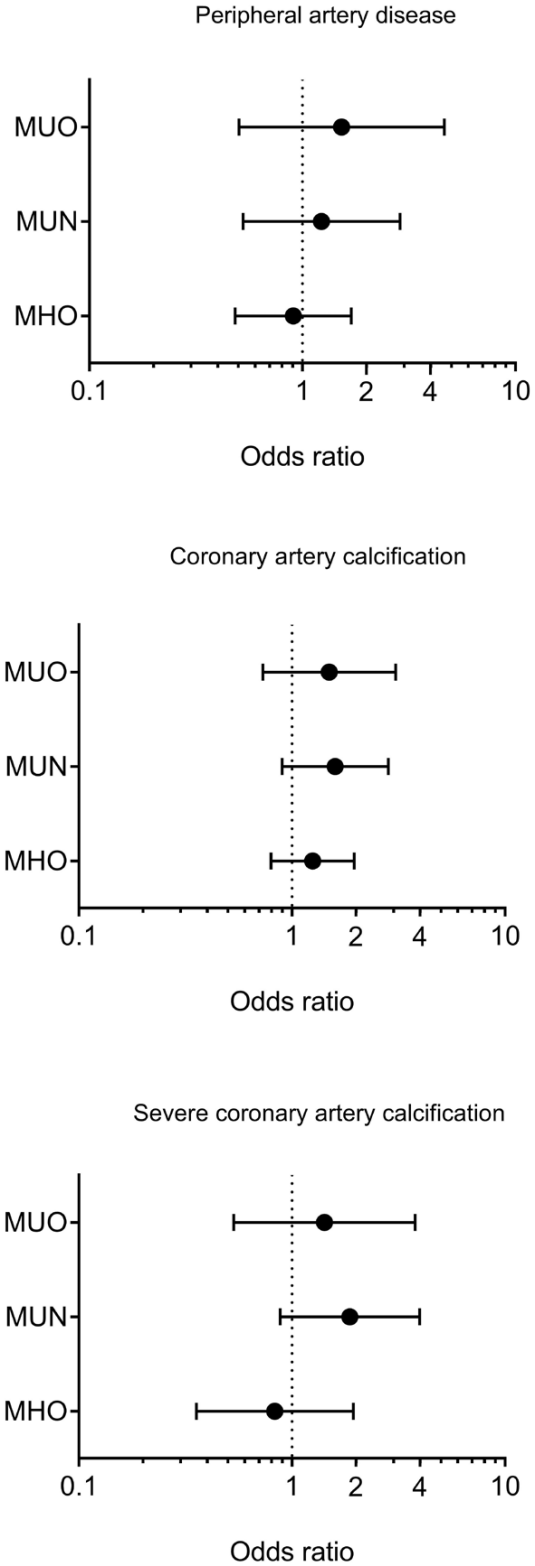
Odds ratios and 95% confidence interval of having macrovascular complications in the four groups with adjustment for age, sex, duration of diabetes, treatment groups in DCCT, and the presence of non‐proliferative retinopathy at baseline. The risk in the MHN group is set as the reference. MHN, metabolically healthy non‐obesity; MHO, metabolically healthy obesity; MUN, metabolically unhealthy non‐obesity; MUO, metabolically unhealthy obesity.

After adjustment for age, sex, duration of diabetes, treatment groups in DCCT and the presence of non‐proliferative retinopathy at baseline, there was no significant difference of odds of having peripheral artery disease, coronary artery calcification or severe coronary artery calcification amongst the four groups (Figure 3).

**FIGURE 4:**
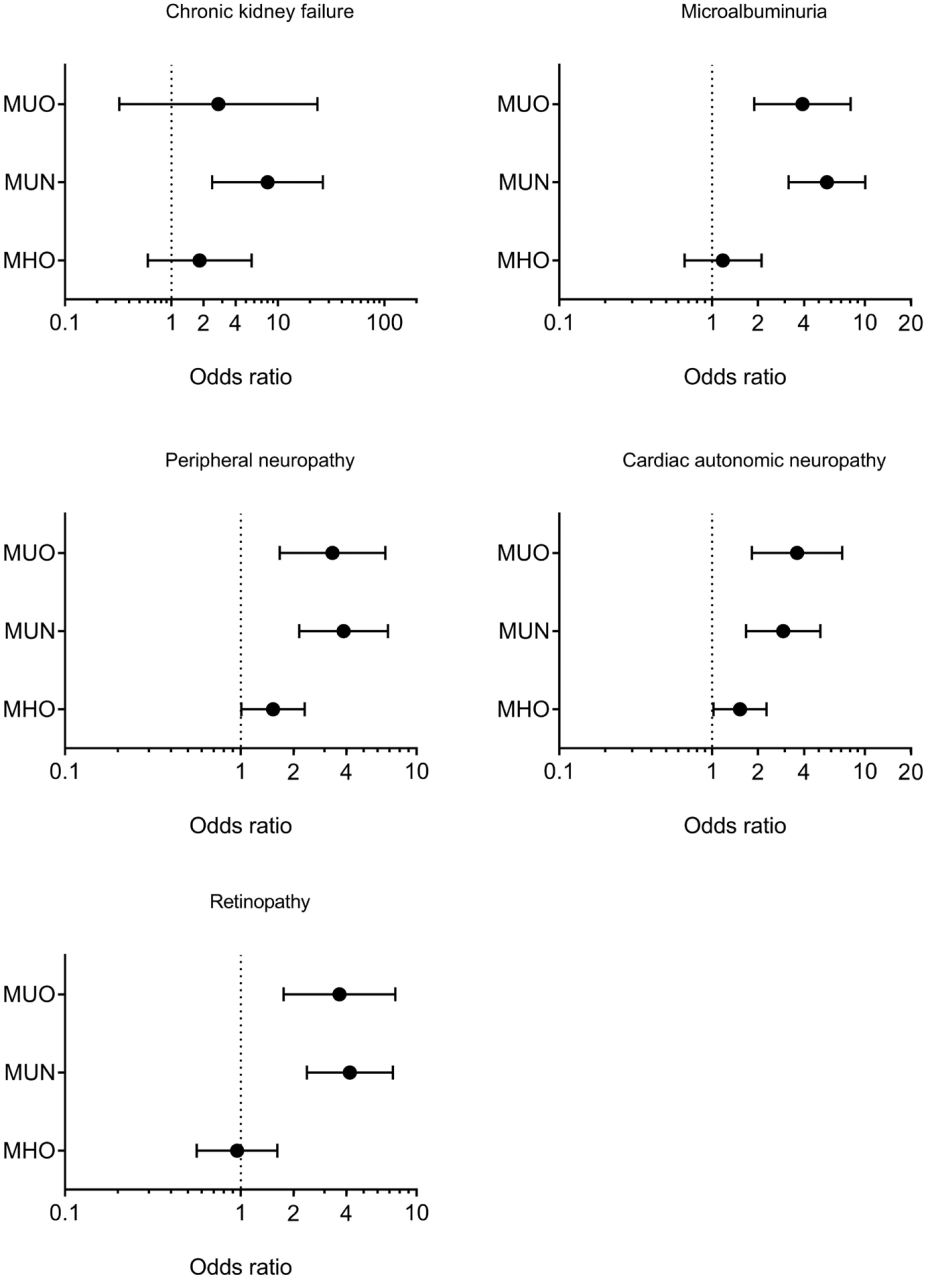
Odds ratios and 95% confidence interval of microvascular complications in the four groups with adjustment for age, sex, duration of diabetes, treatment groups in DCCT, and the presence of non‐proliferative retinopathy at baseline. The risk in the MHN group is set as the reference. MHN, metabolically healthy non‐obesity; MHO, metabolically healthy obesity; MUN, metabolically unhealthy non‐obesity; MUO, metabolically unhealthy obesity.

Notably, after adjustment for age, sex, duration of diabetes, treatment groups in DCCT and the presence of non‐proliferative retinopathy at baseline, the MUO and MUN groups had significantly higher odds of most diabetic microvascular complications than the MHN group (*p* < 0.05; Figure 4). Specifically, the MUO and MUN groups had 3.9 (1.9–8.1) and 5.7 (3.2–10.1) times higher odds (95%CI) of having microalbuminuria, 3.3 (1.7–6.7) and 3.8 (2.2–6.9) times higher odds (95%CI) of having peripheral neuropathy, 3.6 (1.8–7.1) and 2.9 (1.7–5.1) times higher odds (95%CI) of having cardiac autonomic neuropathy, 3.6 (1.8–7.6) and 4.2 (2.4–7.3) times higher odds (95%CI) of having retinopathy compared to the MHN group; whereas the MUN group also had 8.0 (2.4–26.4) times higher odds (95%CI) of having chronic kidney failure compared to the MHN group. There was no difference in odds of having chronic kidney failure, microalbuminuria, and retinopathy between MHO and MHN groups, meanwhile the MHO group had 1.5 (1.0–2.3) and 1.5 (1.0–2.3) times higher odds (95%CI) of having peripheral neuropathy and cardiac autonomic neuropathy than the MHN group (Figure 4).

**FIGURE 5:**
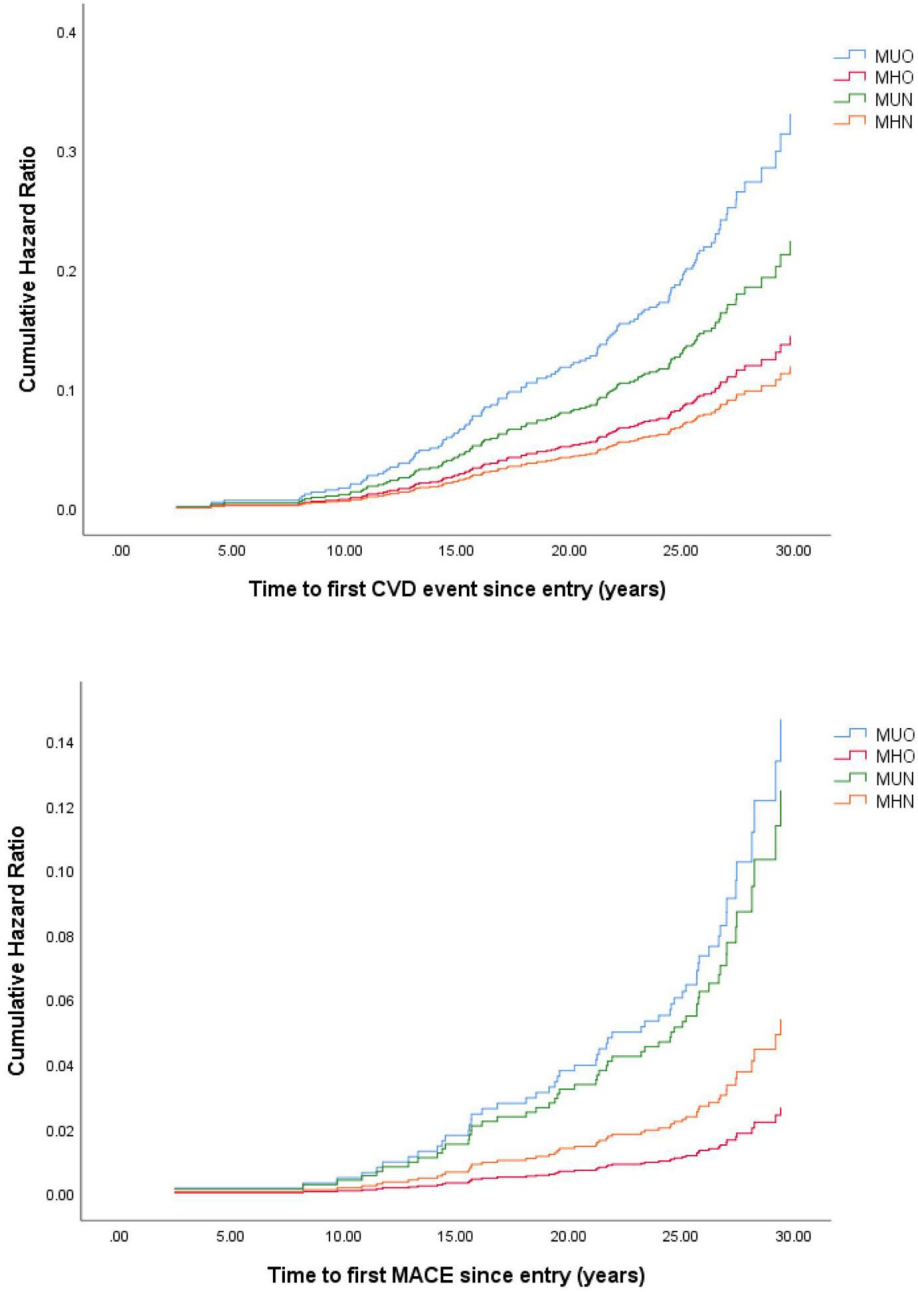
Hazard ratios for any CVD and for MACE in the four groups. After adjustment for age, sex, duration of diabetes, treatment groups in DCCT, and the presence of non‐proliferative retinopathy at baseline, the MUO and MUN groups had higher risks of both any CVD and MACE compared with the MHN group (*p* < 0.05), while the hazard ratios were not different between the MHO and MHN groups. CVD, Cardiovascular events; MACE, Major atherosclerotic cardiovascular events; MHN, metabolically healthy non‐obesity; MHO, metabolically healthy obesity; MUN, metabolically unhealthy non‐obesity; MUO, metabolically unhealthy obesity.

Figure 5 presents HRs (95% CIs) for any CVD and for MACE estimated from Cox proportional hazards models comparing the four groups, with adjustment for age, sex, duration of diabetes, treatment groups in DCCT and the presence of non‐proliferative retinopathy at baseline. The MUO and MUN groups had significantly higher risks of both any CVD (2.78 (1.51–5.11) in the MUO group; 1.88 (1.05–3.36) in the MUN group) and MACE (2.72 (1.16–6.37) in the MUO group; 2.31 (1.05–5.10) in the MUN group) compared with the MHN group (*p* < 0.05), while the HRs for the MHO group (CVD: 1.22 (0.72–2.06); MACE: 0.49 (0.15–1.62)) was not different from those for the MHN group.

## 4 Discussion

In this study, we compared the development of diabetic complications and cardiovascular events in people with T1D by their obesity and metabolic status over 30 years follow up. Our results showed that although people in the MHO group had higher serum CRP levels and lower adiponectin levels than those in the MHN group, the MHO group did not have higher risk of macrovascular complications such as carotid IMT and cardiovascular events than the MHN group. Besides, microvascular complications (except peripheral neuropathy and cardiac autonomic neuropathy) were not different between MHO and MHN groups. However, both MUO and MUN groups had significantly higher risk of peripheral neuropathy, cardiac autonomic neuropathy, retinopathy and microalbuminuria than the MHN group. Furthermore, the MUO and MUN groups also had greater carotid IMT than both the MHO and MHN groups. Most importantly, any CVD and MACE risks were also significantly higher in the MUO and MUN groups than that in the MHO and MHN groups.

There is no universally accepted definition of MHO. Investigators have frequently used either the absence of the metabolic syndrome or insulin resistance (usually calculated by fasting insulin and glucose levels based on several models) to define metabolic health [2]. Insulin resistance is thought to represent one of the most important mechanisms of metabolic diseases [24]. Studies suggested that some people with obesity are not insulin resistant [2, 4]. Furthermore, when MHO was defined as the absence of metabolic syndrome, insulin resistance in this MHO population determined the increased risk for type 2 diabetes [25]. Therefore, the effective way to differentiate individuals with MHO from metabolically at‐risk obese individuals is to assess insulin resistance [2]. Since the fasting insulin levels are negligible in people with T1D, we calculated the insulin resistance by eGDR, which has been used in the T1D studies [10, 15]. A previous study analysed the risk factors for cardiovascular disease in T1D [21]. The results showed hyperglycaemia is an important risk factor second only to age. The study also found significant associations of current eGDR values with any CVD and MACE, but it was diminished after adjustment for age and updated mean HbA1c [21]. The present study used different eGDR values from the previous study. We calculated the eGDR values at baseline and then used the updated mean eGDR in our analyses instead of current eGDR. This might explain the difference in results.

The prevalence of MHO was reported ranging from 6% to 60% in adults with obesity, depending on the criteria used to define metabolic health and the demographics of the studied population [26, 27]. A prior study showed that more than 40% of participants in the National Health and Nutrition Examination Survey III programme were classified as MHO using the National Cholesterol Education Program Adult Treatment Panel III criteria for metabolic syndrome [28], but only 20% fell into the MHO category using more strict insulin sensitivity parameter cutoffs [29]. In the present study, MHO accounts for 78% of people with obesity in T1D which is likely higher than in non‐T1D population although we did not do the comparison. Besides, in the present study, people in the MHO group were more common in women, younger, and having lower BMI than those of the MUO group which is consistent with the non‐TID population [2, 30].

Activation of proinflammatory lymphocytes and expression of inflammation‐related genes in adipose tissue are higher in people with MUO than with MHO [31, 32]. Plasma CRP levels and other inflammatory factors are either higher in those with MUO than with MHO [33, 34, 35] or not different between the two groups [36, 37]. Serum Adiponectin is inversely associated with percentage body fat and directly associated with insulin sensitivity [38]. Plasma adiponectin concentrations are often lower in people with MUO than with MHO [39, 40]. However, in the present study, there was no difference of CRP and adiponectin levels between MHO and MUO groups. This could be related to the specific population with T1D who has autoimmune background and insulin deficiency.

In general, the risks of CVD and all‐cause mortality are greater in people with MUO than in those with MHO and greater in those with MHO than in those with MHN [41, 42, 43, 44, 45]. However, meta‐analysis reported an insignificant association between MHO and cardiovascular events when it limited to studies using the strictest definition for metabolic health (i.e., absence of all metabolic abnormalities) [46]. On the other side, individuals with no obesity can have metabolic abnormalities and be at a high risk of cardiovascular events [47]. Our study also showed that both the MUO and MUN groups had higher risk for any CVD and MACE than the MHN group, but there was no difference of risk for any CVD and MACE between the MHO and MHN groups. These results further highlight the importance of metabolic health represented by insulin resistance in the development of cardiovascular events in people with T1D. Besides, the significant differences of any CVD and MACE between MUN and MHN groups had disappeared after adjustment with statin and Angiotensin‐converting enzyme ever use. The results suggest that the impact of metabolic health on cardiovascular events in people of non‐obesity is partially from hypertension and dyslipidaemia (Tables S2 and S3). Moreover, a previous study in DCCT/EDIC cohort found that intensive therapy for T1D reduced macrovascular events compared with conventional therapy in the first 13 years, even when excessive weight gain occurred. After this, total CVD events significantly increased in the heaviest group with intensive therapy, becoming equivalent to those in the conventional group [48]. In our study, we adjusted the results for intensive therapy, our results further showed metabolic health is as important as weight status changes. Since the previous study did not take insulin resistance into consideration [48], the increased CVD events in the heaviest group with intensive therapy might be related to their insulin resistance.

There is limited available data on microvascular complications in subjects with MHO. Previous studies showed that people with MHO had an increased risk of decline in kidney function but not kidney failure over a median 5‐year period compared with people with MHN [49]. In another study, higher BMI was associated with a lower risk of ESRD amongst those with MHO [50]. Our study did not find any difference in the risk for chronic kidney failure or microalbuminuria between the MHO and MHN groups. Notably, our study first showed that in people with T1D, MHO did increase the risk of peripheral neuropathy and CAN compared to MHN. Conversely, both MUN and MUO groups had significantly higher risk for almost all microvascular complications than the MHN group. A previous study showed higher mean HbA1c and older age were the most significant risk factors for peripheral neuropathy and CAN [51]. Our present study further showed metabolic health represented by eGDR could be an extra risk factor for both clinical peripheral neuropathy and CAN. Moreover, our prior study also showed that the improved insulin resistance over an average of 18.5 years decreased the risk of clinical peripheral neuropathy, whereas the deteriorated insulin resistance increased the risk of diabetic complications including clinical peripheral neuropathy and CAN [17]. Notably, after the adjustment with smoking and medication uses, the significant differences of clinical peripheral neuropathy and CAN between MHO and MHN groups had disappeared; this suggests the impact of obesity to neuropathy in the present cohort is partly explained by smoking, dyslipidaemia or hypertension (Table S1).

Hyperglycaemia is a well‐known strong risk factor for diabetic complications and healthcare providers are routinely targeting it for people with diabetes. In our study, since glycaemia is a part of the eGDR equation, we did not adjust the results for hyperglycaemia. As showed in the Table 1, the time‐weighted mean haemoglobin A1c levels in the two metabolically unhealthy groups were comparable to the two metabolically healthy groups, however, metabolic unhealthy groups did have higher risk of developing diabetic complications. It suggests the importance of metabolic health represented by insulin resistance, which might explain the extra risk for the development of diabetic complications beyond glycaemic control. In summary, our study highlights the importance of metabolic health represented by insulin resistance in the development of diabetic complications and cardiovascular events in a T1D population beyond their weight status. Therefore, it is critical to assess metabolic health in patients with T1D even if they are considered non‐obese. Those who have insulin resistance are more prone to complications and require more intensive and target therapy.

There are some limitations. This is a retrospective analysis, and the casual relationship may be affected by residual confounding. Besides, based on the DCCT/EDIC study protocol, the participants were not typical of the general population with T1D. They were relatively young and healthy without severe complications at entry. They received regular monitoring and more support than the average person with T1D. Therefore, the results could be limited by the source of patients and the generalisability. Moreover, as an indirect estimate of insulin resistance, eGDR is also limited by its sensitivity and specificity by the demography of the cohort, especially it does not put the sex difference into the formula. Lastly, DCCT/EDIC study only measured one‐time concentrations of serum adiponectin and CRP. As with other biochemical markers, serum adiponectin and CRP concentrations may vary over time.

## Author Contributions

Y.M. designed the study, conducted data analysis and wrote the manuscript. J.G. and N.J. reviewed and edited the manuscript.

## Conflicts of Interest

The authors declare no conflicts of interest.

## Supporting information


Data S1.

## Data Availability

The data that support the findings of this study are openly available in Diabetes Control and Complications Trial/Epidemiology of D at https://repository.niddk.nih.gov/studies/edic/.
