# Supplementary material for: Obesity, Metabolic Health, and Diabetic Complications in People With Type 1 Diabetes
Source: Endocrinol Diabetes Metab. 2024 Dec 16;8(1):e70017. doi: 10.1002/edm2.70017 (PMC11648318; doi:10.1002/edm2.70017)
Supplement: Supplementary file 1 — Data S1. [file EDM2-8-e70017-s001.pdf]

**Supplemental Information**

**Supplemental Table 1** Odds ratios of diabetic complications in the four groups.

| | **Odds Ratio** | **95% CI** | **P value** |
|---|---|---|---|
| **Peripheral artery disease** | | | |
| *Model 1* | | | |
| MUN vs. MHN | 1.229 | 0.527- 2.870 | 0.633 |
| MHO vs. MHN | 0.906 | 0.483- 1.698 | 0.757 |
| MUO vs. MHN | 1.529 | 0.505- 4.633 | 0.453 |
| *Model 2* | | | |
| MUN vs. MHN | 0.957 | 0.403- 2.275 | 0.921 |
| MHO vs. MHN | 0.897 | 0.473- 1.699 | 0.738 |
| MUO vs. MHN | 1.276 | 0.414- 3.932 | 0.671 |
| **Coronary artery calcification** | | | |
| *Model 1* | | | |
| MUN vs. MHN | 1.596 | 0.899- 2.833 | 0.111 |
| MHO vs. MHN | 1.251 | 0.797- 1.964 | 0.329 |
| MUO vs. MHN | 1.498 | 0.731- 3.070 | 0.270 |
| *Model 2* | | | |
| MUN vs. MHN | 1.291 | 0.709- 2.352 | 0.403 |
| MHO vs. MHN | 1.339 | 0.845- 2.123 | 0.214 |
| MUO vs. MHN | 1.361 | 0.656- 2.824 | 0.407 |
| **Severe coronary artery calcification** | | | |
| *Model 1* | | | |
| MUN vs. MHN | 1.870 | 0.880- 3.974 | 0.104 |
| MHO vs. MHN | 0.831 | 0.356- 1.940 | 0.669 |
| MUO vs. MHN | 1.422 | 0.533- 3.791 | 0.482 |
| *Model 2* | | | |
| MUN vs. MHN | 1.432 | 0.642- 3.194 | 0.381 |
| MHO vs. MHN | 0.927 | 0.388- 2.217 | 0.865 |
| MUO vs. MHN | 1.307 | 0.476- 3.589 | 0.603 |

| | | | |
|---|---|---|---|
| **Microalbuminuria** | | | |
| *Model 1* | | | |
| MUN vs. MHN | 5.655 | 3.177- 10.065 | < 0.001 |
| MHO vs. MHN | 1.178 | 0.660- 2.105 | 0.579 |
| MUO vs. MHN | 3.905 | 1.888- 8.076 | <0.001 |
| *Model 2* | | | |
| MUN vs. MHN | 3.161 | 1.737- 5.755 | < 0.001 |
| MHO vs. MHN | 0.980 | 0.535- 1.796 | 0.949 |
| MUO vs. MHN | 2.623 | 1.233- 5.580 | 0.012 |
| | | | |
| **Chronic kidney failure** | | | |
| *Model 1* | | | |
| MUN vs. MHN | 7.976 | 2.409- 26.414 | 0.001 |
| MHO vs. MHN | 1.840 | 0.600- 5.650 | 0.414 |
| MUO vs. MHN | 2.760 | 0.324- 23.496 | 0.441 |
| *Model 2* | | | |
| MUN vs. MHN | 3.615 | 1.121- 11.662 | 0.031 |
| MHO vs. MHN | 0.339 | 0.041- 2.792 | 0.315 |
| MUO vs. MHN | 1.428 | 0.166- 12.253 | 0.745 |
| | | | |
| **Clinical peripheral neuropathy** | | | |
| *Model 1* | | | |
| MUN vs. MHN | 3.848 | 2.153- 6.878 | < 0.001 |
| MHO vs. MHN | 1.528 | 1.009- 2.313 | 0.045 |
| MUO vs. MHN | 3.327 | 1.664- 6.651 | 0.001 |
| *Model 2* | | | |
| MUN vs. MHN | 2.903 | 1.604- 5.250 | < 0.001 |
| MHO vs. MHN | 1.461 | 0.958- 2.228 | 0.079 |
| MUO vs. MHN | 2.622 | 1.298- 5.296 | 0.007 |
| | | | |
| **Cardiac Autonomic neuropathy** | | | |
| *Model 1* | | | |

| | | | |
|---|---|---|---|
| MUN vs. MHN | 2.924 | 1.672- 5.111 | <0.001 |
| MHO vs. MHN | 1.526 | 1.023- 2.275 | 0.038 |
| MUO vs. MHN | 3.604 | 1.825- 7.115 | <0.001 |
| *Model 2* | | | |
| MUN vs. MHN | 1.974 | 1.111- 3.507 | 0.020 |
| MHO vs. MHN | 1.441 | 0.953- 2.178 | 0.083 |
| MUO vs. MHN | 2.822 | 1.407- 5.662 | 0.003 |
| | | | |
| **Retinopathy** | | | |
| *Model 1* | | | |
| MUN vs. MHN | 4.176 | 2.378- 7.333 | <0.001 |
| MHO vs. MHN | 0.953 | 0.562- 1.615 | 0.858 |
| MUO vs. MHN | 3.645 | 1.753- 7.579 | 0.001 |
| *Model 2* | | | |
| MUN vs. MHN | 3.060 | 1.711- 5.471 | <0.001 |
| MHO vs. MHN | 0.861 | 0.503- 1.472 | 0.584 |
| MUO vs. MHN | 2.840 | 1.352- 5.969 | 0.006 |

Odds ratios and 95% confidence interval of having diabetic complications in the four groups with adjustment for age, sex, duration of diabetes, intensive treatment in DCCT, and the presence of non-proliferative retinopathy at baseline in the model 1. Model 2 is additionally adjusted for smoking at entry, ever smoking, statin ever use, ACE inhibitor ever use, and ARB ever use. The risk in the MHN group is set as the reference. ACE, Angiotensin-converting enzyme; ARB, Angiotensin II receptor blockers; MHN, metabolically healthy non-obesity; MHO, metabolically healthy obesity; MUN, metabolically unhealthy non-obesity; MUO, metabolically unhealthy obesity.

**Supplemental Table 2** Hazard ratios for any CVD in the four groups in the Cox proportional hazards regression models.

| | Original Model (in the manuscript) | | | Model further included smoking and medication uses | | |
|---|---|---|---|---|---|---|
| | HR | 95% CI | P | HR | 95% CI | P |
| Age (years) | 1.091 | 1.061-1.122 | <0.001 | 1.076 | 1.045-1.108 | <0.001 |
| Female vs. male | 1.299 | 0.907-1.861 | 0.153 | 1.402 | 0.979-2.009 | 0.065 |
| Duration of diabetes | 0.999 | 0.995-1.004 | 0.780 | 0.999 | 0.995-1.004 | 0.809 |
| Intensive Treatment in DCCT | 0.717 | 0.503-1.021 | 0.065 | 0.734 | 0.513-1.051 | 0.091 |
| Presence of non-proliferative retinopathy at baseline | 1.616 | 0.983-2.655 | 0.58 | 1.579 | 0.956-2.583 | 0.069 |
| MUN vs. MHN | 1.883 | 1.054-3.362 | 0.032 | 1.350 | 0.751-2.424 | 0.316 |
| MHO vs. MHN | 1.215 | 0.716-2.062 | 0.471 | 1.215 | 0.711-2.077 | 0.477 |
| MUO vs. MHN | 2.778 | 1.510-5.111 | 0.001 | 2.375 | 1.283-4.396 | 0.006 |
| Smoking at entry | | | | 1.201 | 0.672-2.145 | 0.537 |
| Ever smoking | | | | 1.504 | 0.889-2.544 | 0.128 |
| Statin ever use | | | | 3.246 | 1.486-7.090 | 0.003 |
| ACE inhibitor ever use | | | | 2.541 | 1.393-4.635 | 0.002 |
| ARB ever use | | | | 0.948 | 0.661-1.360 | 0.772 |

The risk in the MHN group is set as the reference. ACE, Angiotensin-converting enzyme; ARB, Angiotensin II receptor blockers; CVD: cardiovascular events; MACE: major atherosclerotic cardiovascular events; MHN, metabolically healthy non-obesity; MHO, metabolically healthy obesity; MUN, metabolically unhealthy non-obesity; MUO, metabolically unhealthy obesity.

**Supplemental Table 3** Hazard ratios for MACE in the four groups in the Cox proportional hazards regression models.

| | Original Model (in the manuscript) | | | Model further included smoking and medication uses | | |
|---|---|---|---|---|---|---|
| | HR | 95% CI | P | HR | 95% CI | P |
| Age (years) | 1.111 | 1.062-1.163 | <0.001 | 1.088 | 1.038-1.140 | <0.001 |
| Female vs. Male | 0.921 | 0.523-1.623 | 0.777 | 0.990 | 0.563-1.741 | 0.972 |
| Duration of diabetes | 0.998 | 0.991-1.005 | 0.570 | 0.998 | 0.991-1.005 | 0.626 |
| Intensive Treatment in DCCT | 0.803 | 0.463-1.392 | 0.434 | 0.779 | 0.445-1.365 | 0.383 |
| Presence of non-proliferative retinopathy at baseline | 1.801 | 0.829-3.912 | 0.137 | 1.736 | 0.803-3.751 | 0.161 |
| MUN vs. MHN | 2.314 | 1.051-5.096 | 0.037 | 1.653 | 0.745-3.668 | 0.217 |
| MHO vs. MHN | 0.493 | 0.150-1.620 | 0.244 | 0.543 | 0.163-1.810 | 0.320 |
| MUO vs. MHN | 2.721 | 1.162-6.370 | 0.021 | 2.695 | 1.133-6.410 | 0.025 |
| Smoking at entry | | | | 2.366 | 0.874-6.405 | 0.090 |
| Ever smoking | | | | 1.159 | 0.438-3.064 | 0.766 |
| Statin ever use | | | | 8.256 | 1.118-60.945 | 0.038 |
| ACE inhibitor ever use | | | | 2.407 | 0.920-6.297 | 0.073 |
| ARB ever use | | | | 0.700 | 0.395-1.240 | 0.221 |

The risk in the MHN group is set as the reference. ACE, Angiotensin-converting enzyme; ARB, Angiotensin II receptor blockers; CVD: cardiovascular events; MACE: major atherosclerotic cardiovascular events; MHN, metabolically healthy non-obesity; MHO, metabolically healthy obesity; MUN, metabolically unhealthy non-obesity; MUO, metabolically unhealthy obesity.
